# Feasibility of a portable optical coherence tomography system in children with craniosynostosis

**DOI:** 10.1038/s41433-022-02205-0

**Published:** 2022-08-29

**Authors:** Ravi Purohit, Sohaib R. Rufai, Chetan Khantibai Patel, Gregory P. L. Thomas, Noor ul Owase Jeelani, David Johnson, Tim P. Lawrence

**Affiliations:** 1 Oxford Craniofacial Unit, Oxford University Hospitals NHS Foundation Trust, John Radcliffe Hospital, Oxford, UK; 2 Oxford Eye Hospital, Oxford University Hospitals NHS Foundation Trust, John Radcliffe Hospital, Oxford, UK; 3 Nuffield Department of Clinical Neurosciences, University of Oxford, Oxford, UK; 4 The University of Leicester Ulverscroft Eye Unit, Leicester Royal Infirmary, Leicester, UK; 5 Craniofacial Unit, Great Ormond Street Hospital for Children NHS Foundation Trust and UCL GOS Institute of Child Health, London, UK

**Keywords:** Medical research, Biomarkers

Craniosynostosis is characterised by the premature fusion of cranial sutures and can be associated with intracranial hypertension (IH), which can damage the brain and vision if left unaddressed [1]. The gold standard method for measuring intracranial pressure (ICP) is invasive ICP monitoring, requiring hospital admission, general anaesthesia and risks such as bleeding and infection [2]. Optical coherence tomography (OCT) is a non-invasive imaging technique providing ultra-high resolution cross-sectional images of the optic nerve within seconds. OCT has demonstrated promise as a non-invasive surrogate marker for IH, but standard table-mounted OCT devices are difficult to use in infants and young children [3]. Here, we assessed the feasibility of a portable OCT device (Fig. 1) in children with craniosynostosis.

**Fig. 1:**
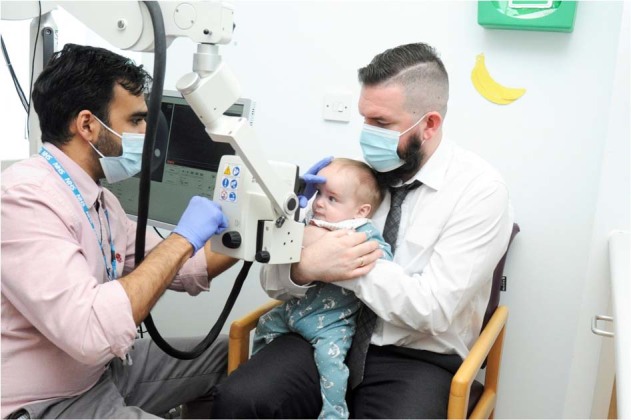
Image acquisition. Spectralis Flex portable OCT device used in infant with craniosynostosis.

This was a multi-centre feasibility study. Fifty children aged under 18 years with a clinical and/or genetic diagnosis of craniosynostosis were included. A portable OCT system was used (Spectralis Flex, Heidelberg Engineering, Heidelberg, Germany). Success rates were recorded for the bilateral and unilateral acquisition of optic nerve head (ONH) images of analysable quality. This was defined as an ONH image where the edges of the disc margins and the cup profile, including its lowermost point, were clearly visualised. This study was approved by Oxford University Hospitals Audit Department (registration ID 5956).

Of the fifty children included in this study, 47 were imaged at the Oxford Craniofacial Unit in outpatient clinics, while three were imaged at Great Ormond Street Hospital (GOSH) on the recovery ward during invasive ICP monitoring. 28 patients were male (56%), 22 were female (44%), 12 had syndromic craniosynostosis (24%), 38 had non-syndromic craniosynostosis (76%). Median age was 47 months (range: 2–173; IQR: 11–80). All 50 patients were awake during imaging, and all OCT images were taken with an assistant operating the computer. No patients were dilated. Bilateral OCT imaging was successful in 39 (78%) and unilateral OCT imaging successful in 44 (88%). The reason for non-acquisition was poor patient compliance in all cases.

To the best of our knowledge, this is the first study to demonstrate that portable OCT imaging using the Spectralis Flex system is feasible in children with craniosynostosis. The success rates for bilateral and unilateral ONH imaging were similar to those recently obtained at Great Ormond Street Hospital (GOSH), using a handheld OCT device [4], and higher than that reported by Patel et al. [5] in a large normative study of children without eye disease (*n* = 351). This study carried the limitations expected of a feasibility study. Only three patients at GOSH underwent OCT imaging using the Spectralis Flex, because the handheld device (Envisu C2300, Leica Microsystems, Wetzlar, Germany) and table-mounted OCT (Spectralis) are predominantly used in these patients at GOSH––interestingly, all three patients had successful bilateral OCT scans during invasive ICP monitoring. The feasibility of OCT during ICP monitoring permits further prospective work to determine the optimal OCT parameters to maximise sensitivity and specificity for IH in craniosynostosis, and to inform clinical guidelines.

## Data Availability

The datasets generated and/or analysed during the current study are available from the corresponding author on reasonable request.
